# Supplementary material for: Development of a computer-aided design software for the quantitative evaluation of aesthetic damage
Source: PLoS One. 2019 Dec 18;14(12):e0226322. doi: 10.1371/journal.pone.0226322 (PMC6919621; doi:10.1371/journal.pone.0226322)
Supplement: S1 Code site — 1. (ZIP) [file pone.0226322.s001.zip › [EESC_jr][Projeto]Relatorio_07_21/vendors/select2/docs/_includes/nav/examples.html]

- The basics
  - Single select boxes
  - Multiple select boxes
- Placeholders
- Data sources
  - Loading array data
  - Loading remote data
- Disabled mode
- Disabled results
- Limiting the number of selections
- Hiding the search box
- Programmatic control
  - DOM events
  - Programmatic access
- Tagging support
- Automatic tokenization
- Customizing how results are matched
- Localization, RTL and diacritics support
  - Multiple languages
  - RTL support
  - Diacritics support
- Themes, templating and responsive design
  - Theme support
  - Templating
  - Responsive design

Back to top
